# Independent corroboration of monitor unit calculations performed by a 3D computerized planning system

**DOI:** 10.1120/jacmp.v1i4.2633

**Published:** 2000-09-01

**Authors:** Konrad W. Leszczynski, Peter B. Dunscombe

**Affiliations:** ^1^ Department of Medical Physics Northeastern Ontario Regional Cancer Centre 41 Ramsey Lake Road Sudbury P3E 5J1 Canada; ^2^ Department of Radiology University of Ottawa Ottawa K1N 6N5 Canada; ^3^ Department of Physics Laurentian University Sudbury Canada P3E 2C6

**Keywords:** monitor unit calculations, quality assurance, treatment planning

## Abstract

The checking of monitor unit calculations is recognized as a vital component of quality assurance in radiotherapy. Using straightforward but detailed computer‐based verification calculations it is possible to achieve a precision of 1% when compared with a three‐dimensional (3D) treatment planning system monitor unit calculation. The method is sufficiently sensitive to identify significant errors and is consistent with current recommendations on the magnitude of uncertainties in clinical dosimetry. Moreover, the approach is accurate in the sense of being highly consistent with the validated 3D treatment planning system's calculations.

PACS number(s): 87.53.–j, 87.52.–g

## INTRODUCTION

The success of radiation therapy crucially depends on the accuracy with which the prescribed dose is delivered to the tumor volume. Quantitative assessments of this relationship have led to the development of recommendations as to the accuracy required in dose delivery. According to the generally accepted recommendations made by the International Commission on Radiation Units and Measurements^1^ the dose delivered should not deviate by more than ±5% from the dose prescribed. More recently Mijnheer *et al*.^2^ and Wambersie *et al*.^3^ proposed that the standard deviation of the uncertainty in the delivered dose should not be greater than 3.5%. As only a part of the overall uncertainty arises from the process of dose calculations in treatment planning, the tolerances for the accuracy of treatment planning systems (TPS) have to be appropriately smaller.

Uncertainties in dose delivery may be introduced at the treatment phase (including machine calibration) or during the process of deriving monitor unit (or timer) settings from the dose prescription determined by the radiation oncologist (treatment preparation). Dose errors of the latter type, arising at the treatment planning phase, could potentially affect the whole course of treatment and therefore are of particular concern. For computerized calculations of monitor units (MU), whether or not accompanied by a dose distribution, uncertainties may be further categorized as arising from the input beam data, the calculation algorithm, incorrect use of the system, and data transfer to the treatment sheet. Although there is a possibility that a significant dose error may arise as a result of beam data or the algorithm, commissioning is designed to minimize this risk.^4^ In routine clinical practice, more likely sources of systematic dose error for individual patients result from misuse of the system (e.g., through inadequate understanding of normalization protocols), misinterpretation of the system output and data transfer errors.

Currently, MU settings required to deliver the prescribed dose are often calculated by a computerized treatment planning system using methods and quantities different from those used in manual MU calculations. This is particularly true of those planning systems, which employ the convolution‐superposition method in their calculations. Published recommendations for quality assurance (QA) in radiation therapy stipulate routine checking of MU calculations using means independent from the original calculator.^5^ ^–^ ^7^ These checks are primarily intended to identify dose errors resulting from the three sources listed above and prior to the start of treatment. Independent checking, as we shall show below, can also enhance confidence in the accuracy of the algorithm and integrity of the beam data used although this is not generally the main intention.

Previously published reports on independent checks of MU calculations^8^ ^–^ ^10^ confirm the usefulness of this QA procedure in promoting the accurate delivery of the prescribed dose. They also provide an indication of the limitations of applicability of conventional dose calculation algorithms employed in computerized treatment planning systems.^10^ In the selection of a method for checking monitor unit calculations it is clearly necessary to establish that the method has sufficient sensitivity, quantified as accuracy and precision, to perform its intended function.

In this paper we describe our experience with an approach to the routine verification of the monitor units calculated by a three‐dimensional (3D) computerized treatment planning system (Helax‐TMS, Helax AB, Uppsala, Sweden). The approach was computer based and incorporated almost all the factors affecting photon dosimetry, which are features of the full (3D) system. The method has been employed for the verification of the monitor unit calculations associated with close to 500 computerized treatment plans. Our analysis enables us to quantify the accuracy and precision of our method, by treatment site, and hence discuss its applicability in routine quality control of computerized monitor unit calculations.

## METHODS AND MATERIALS

The quality control protocol at our cancer center requires all computerized treatment plans to be checked independently by a medical physicist. The study described here is based on timer setting and MU calculation checks performed for 497 treatment plans for patients who were treated with Cobalt‐60, 6‐MV, and 23‐MV photon beams. All common treatment sites were represented in this sample.

Our approach to the checking of Helax‐TMS MU calculations has been based on the standard system of dosimetric calculations, using output factor and tissue phantom ratio (TPR) tables and wedge and tray transmission factors. These factors were acquired totally independently of, and several years prior to, the data used by Helax‐TMS and their applicability verified by analysis of routine QA on our treatment units, which indicated no significant change in radiation beam characteristics throughout the years. Certain simplifications were applied such as the use of a single scatter (output) factor without separating it into the collimator and phantom scatter components.

All cases in this study involve isocentric dose calculations, and the reference (normalization) point for which the MU calculations were checked was defined according to the International Commission on Radiation Units and Measurements (ICRU) 50 recommendations.^12^ In the great majority of cases the reference point was placed at the isocenter. In order to calculate monitor units the Helax‐TMS planning system requires the user to specify the prescribed dose and the percentage isodose level to which it applies. Helax‐TMS will then compute the monitor units for each beam taking into account beam modifiers such as blocks, trays, and wedges. The monitor units so calculated are entered into the patient's chart.

The MU checking method employed an MS‐Excel™ spreadsheet for the dose calculations. The depths for TPRs were measured independently, with a ruler, in the computed technology (CT) section or contours used in planning. The nominal field size was adjusted in the direction parallel to the central section of the treated volume to take into account additional shielding and/or the fact that a part of the field might have fallen outside the patient's body. Density corrections based on radiological depths and TPR ratios were applied only for lung and major bone (e.g., pelvis) inhomogeneities. Dose calculations for the majority of treatment sites were based on CT data. In these cases the Helax‐TMS system derived electron densities from CT numbers through an appropriate calibration. Three major treatment sites for which CT data were not used in dose calculations included the breast/chest wall, supraclavicular node region in breast cancer patients, and rectum. The only tissue inhomogeneity considered in these sites was due to lungs in tangential breast irradiations. A standard value of 0.26 was assigned as the relative electron density within contours representing the lung. In manual calculations always standard rather than CT‐derived values for relative electron densities were used for the major inhomogeneities that were considered. Lungs were assigned the same relative electron density as in non‐CT based TPS calculations and bone inhomogeneities were assumed to have a uniform density of 1.5 which falls within the range defined by the densities of cancellous (spongy) and compact bones. For a wedged field where the reference point was placed off the central axis a correction based on the previously measured dose profiles along the wedged direction was applied. The appropriate dosimetric factors were read from tables, interpolated for the actual field size, and entered manually into the spreadsheet. Thus the general formula used to calculate the dose contribution, *D*, from one field with the monitor unit setting, MU, was as follows:

(1) $$D=K\times \text{MU}\times S_{c,p}\times \text{TPR}\times \text{ISqF}\times \text{TF}\times \text{WTF}\times \text{OAF}\times \text{CF}.$$

In the above equation, *K* is the output factor (cGy/MU) under calibration conditions; $S_{c,p}$ denotes the scatter (output) factor; ISqF is an inverse‐square correction factor applied when nonstandard treatment distance was used; TF and WTF are the shielding/compensator tray and wedge transmission factors, respectively; OAF is the off‐axis correction factor, used in off‐axis calculations for wedged fields only; and CF represents a correction factor for tissue inhomogeneities.

The analysis below consisted of taking the ratio of the independently evaluated dose to the reference point to that specified by the user in Helax‐TMS and generating the means and standard deviations by site for the check method.

## RESULTS AND DISCUSSION

**Figure 1:**
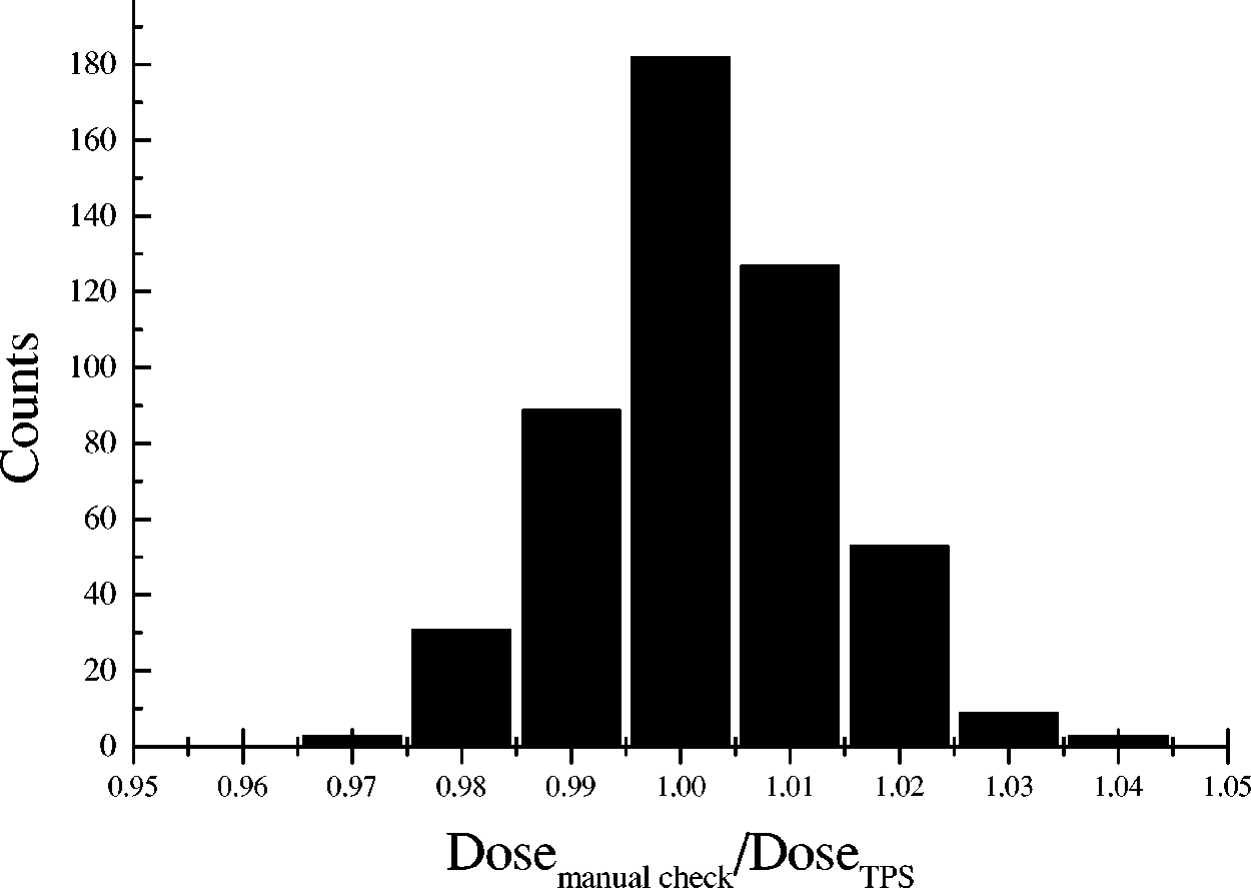
A histogram showing the distribution of the ratios of independently evaluated reference point doses to those calculated by Helax‐TMS.

The histogram in Fig. 1 shows frequency counts of the ratios of independently evaluated reference point doses to those calculated by Helax‐TMS. Table I contains the results of the statistical analysis of these data. The 497 cases included have been divided into eight anatomical sites for which the averages and standard deviations of the dose ratios are tabulated. The different anatomical sites represent different degrees of complexity of dose calculations performed both manually and by Helax‐TMS, in terms of inhomogeneities, contour and field shape irregularities, and off‐axis corrections.

**Table I:** The averages and standard deviations (SD) of the ratios of independently evaluated reference point doses to those calculated by Helax‐TMS. The results are grouped according to the major anatomical sites.

Site group	*n*	Average	SD
*Brain*	23	1.007	0.006
*Head & neck*	83	1.006	0.012
*Lung/esophagus*	44	1.009	0.014
*Breast/chest wall*	117	1.001	0.009
*Supraclavicular*	24	1.013	0.010
*Abdomen/pelvis* ^a^	37	1.003	0.013
*Prostate*	110	1.002	0.008
*Rectum*	59	0.990	0.009
*All sites*	497	1.002	0.012

a Excluding prostate and rectum, which are analyzed separately.

From the results shown in Table I the limiting standard deviation appears to be of the order of 1%. The sites for which standard deviations are smaller are those where the dose calculation geometry, in terms of external contours, internal inhomogeneities, and beam orientations, is very consistent from case to case. This is especially true for the two most common treatment sites, i.e., breast and prostate as well as some less common ones such as brain and rectum. Geometric variability in internal tissue inhomogeneities, beam orientations with respect to external contours and in the position of the dose reference points lead to somewhat poorer precision for manual calculations performed for lung, abdomen/pelvis, and head and neck cases, respectively.

The accuracy of the MU check method can be inferred from the agreement between the manual calculations and those performed by the 3D system. The Helax‐TMS treatment planning system has been extensively validated using several techniques including thermoluminescence dosimetry (TLD) measurements in an anthropomorphic phantom^4^ and is believed to be accurate. In nearly 500 plans the reference point dose calculated is, on the average, only 0.2% different from that specified in the computerized treatment plan for MU calculations. This overall consistency between the MU check method and full 3D calculations leads us to believe that the check method is also highly accurate. It should be noted, however, that the accuracy of the manual MU calculations varies somewhat with the treatment site. The largest numerically and statistically most significant systematic deviations exist in calculations for supraclavicular and rectum treatment plans. In the case of supraclavicular calculations this is attributable to not taking into account off‐axis dose profile variations in the half‐blocked supraclavicular fields (the only off‐axis correction applied was due to wedge). The systematic deviation of 1% in the case of rectum plans is primarily due to ignoring in manual MU checks the beam hardening effect produced by wedge filters. This effect is not pronounced at shallower depths in other treatment sites where wedges are used (e.g., breast). The dose underestimation due to neglecting the beam hardening effect is also not present in calculations for treatment sites of similar geometry to that of the rectum, but where wedge filters were not used (e.g., prostate). The third significant systematic inaccuracy that can be identified in the results occurred in the calculations for lung and esophagus. It can be explained by the fact that the manual calculation method did not take into account scatter dose perturbations caused by tissue inhomogeneities. These systematic effects are very small and, in the opinion of the authors, are insignificant in the context of an MU checking procedure. However, if desired, they could be simply corrected for by applying a site dependent offset deduced from the results presented in Table I.

The appropriateness of these methods for routine quality control of monitor units calculated by 3D computerized treatment planning computers can be assessed by comparing the accuracy and precision results of Table I with available recommendations and analyses. Clearly if these two statistical characteristics are inconsistent with the sensitivity required of a checking procedure then the method is of little value. Van Dyk *et al*.^13^ suggest that the accuracy of a treatment planning system should be 4% in a low dose gradient region with all corrections invoked. Mijnheer *et al*.^2^ have quantitatively analyzed the sources of uncertainty in clinical dosimetry. Their analysis suggests that the treatment planning process may introduce an uncertainty of 2–3%. Both of these recommendations presumably refer to the agreement between the calculated dose at a point with that that would be measured under the same irradiation conditions at the same point. As such, these recommendations are not directly transferable to monitor unit check procedures, which compare one method of calculation with another, although they do provide a context within which to suggest the minimum sensitivity. Ideally, a monitor unit check procedure should provide a reasonable guarantee that the recommended uncertainties^2^ ^,^ ^13^ are the limiting uncertainties in the treatment preparation phase of a course of radiotherapy. Thus errors which the checking procedure can identify, *viz*. system misuse, output misinterpretation, and data transfer, should not, if present, result in a significant increase in the potential discrepancy between intended and delivered dose. Such a criterion will be met if, as with the checking procedure described here, the accuracy and precision of the method are numerically less than the same intrinsic characteristics of the treatment planning system.^2^ ^,^ ^13^

The usefulness of independent checks of MU calculations is generally recognized and this study indicated that in typical situations it is possible to corroborate the calculations of dose to a reference point, performed by an advanced 3D planning system, using standard methods of manual dose calculation. At the same time one expects that with the advent of modern treatment techniques involving complex noncoplanar treatment geometries, intensity‐modulated and dynamic irradiations, it will become increasingly difficult to check MU calculations using the traditional manual approach. As a possible solution, development of a standardized computerized system for verification of MU calculations has been suggested by the European Society for Therapeutic Radiology and Oncology (ESTRO) MU working group.^14^ It is also expected that *in vivo* dosimetry will be playing an increasing role in verification of dose calculation in advanced 3D conformal treatments^15^ where accurate manual calculations are not possible.

## CONCLUSION

Acceptable sensitivity for the verification of monitor unit calculations performed by a 3D treatment planning system can be achieved, with care, using the standard system of dosimetric calculations.

## ACKNOWLEDGMENTS

The authors wish to thank Sonja Desjardins for her assistance. The generous support of the Northern Cancer Research Foundation is also gratefully acknowledged.

## References

[1] International Commission on Radiation Units and Measurements (ICRU) , Determination of absorbed dose in a patient irradiated by beams of X or gamma rays in radiotherapy procedures, ICRU Report 24 (ICRU Publications, Washington, D.C., 1976).

[2] B. J. Mijnheer , J. J. Battermann , and A. Wambersie , “What degree of accuracy is required and can be achieved in photon and neutron therapy?,” Radiother. Oncol. 8, 237–252 (1987). 3107087 10.1016/s0167-8140(87)80247-5

[3] A. Wambersie , J. Van Dam , G. Hanks , B. J. Mijnheer , and J. J. Battermann , “What accuracy is needed in dosimetry?,” IAEA–TECDOC 734, 11–35 (1994).

[4] P. Dunscombe , P. McGhee , and E. Lederer , “Anthropomorphic phantom measurements for the validation of a treatment planning system,” Phys. Med. Biol. 41, 399–411 (1996). 8778822 10.1088/0031-9155/41/3/005

[5] G. Kutcher *et al*, “Comprehensive QA for radiation oncology: Report of AAPM Radiation Therapy Committee Task Group 40,” Med. Phys. 21, 581–618 (1994). 8058027 10.1118/1.597316

[6] B. Fraass *et al*, “AAPM Radiation Therapy Committee Task Group 53: Quality assurance for clinical radiotherapy treatment planning,” Med. Phys. 25, 1773–1829 (1998). 9800687 10.1118/1.598373

[7] A. Dutreix , B. R. Bjarngard , A. Bridier , B. Mijnheer , J. E. Shaw , and H. Svensson , Monitor Unit Calculation for High Energy Photon Beams (Garant, Leuven, 1997).

[8] R. A. Dahl , E. C. McCullough , and D. E. Mellenberg , “A quality assurance program for monitor unit calculators,” Med. Phys. 17, 103–105 (1990). 2308540 10.1118/1.596539

[9] L. Duggan , T. Kron , S. Howlett , A. Skov , and P. O'Brien , “An independent check of treatment plan, prescription and dose calculation as a QA procedure,” Radiother. Oncol. 42, 297–301 (1997). 9155082 10.1016/s0167-8140(97)01906-3

[10] R. F. Hill , M. D. Perez , and W. A. Beckham , “An independent check method of radiotherapy computer plan derived units,” Australas. Phys. Eng. Sci. Med. 21, 79–84 (1998). 9745794

[11] F. M. Khan , The Physics of Radiation Therapy, 2nd ed. (Williams and Wilkins, Baltimore, 1994).

[12] International Commission on Radiation Units and Measurements (ICRU) , Prescribing, recording, and reporting photon beam therapy, ICRU Report 50 (ICRU, Bethesda, MD, 1993).

[13] J. Van Dyk , R. B. Barnett , J. E. Cygler , and P. C. Schragge , “Commissioning and quality assurance of treatment planning computers,” Int. J. Radiat. Oncol., Biol., Phys. 26, 261–273 (1993). 8491684 10.1016/0360-3016(93)90206-b

[14] J. Olofsson , D. Georg , M. Karlsson , A. Dutreix , and H. Svensson , “Quality assurance of monitor unit calculations (abstract),” Radiother. Oncol. 51, Suppl. 1, 26 (1999).

[15] M. Essers and B. J. Mijnheer , “In vivo dosimetry during external photon beam radiotherapy,” Int. J. Radiat. Oncol., Biol., Phys. 43, 245–259 (1999). 10030247 10.1016/s0360-3016(98)00341-1

